# Early Life Stress Alters Gene Expression and Cytoarchitecture in the Prefrontal Cortex Leading to Social Impairment and Increased Anxiety

**DOI:** 10.3389/fgene.2021.754198

**Published:** 2021-11-02

**Authors:** Noriyoshi Usui, Yuta Ono, Ryoko Aramaki, Stefano Berto, Genevieve Konopka, Hideo Matsuzaki, Shoichi Shimada

**Affiliations:** ^1^ Department of Neuroscience and Cell Biology, Graduate School of Medicine, Osaka University, Suita, Japan; ^2^ United Graduate School of Child Development, Osaka University, Suita, Japan; ^3^ Global Center for Medical Engineering and Informatics, Osaka University, Suita, Japan; ^4^ Addiction Research Unit, Osaka Psychiatric Research Center, Osaka Psychiatric Medical Center, Osaka, Japan; ^5^ Division of Development of Mental Functions, Research Center for Child Mental Development, University of Fukui, Fukui, Japan; ^6^ Department of Neuroscience, Medical University of South Carolina, Charleston, SC, United States; ^7^ Department of Neuroscience, University of Texas Southwestern Medical Center, Dallas, TX, United States; ^8^ Life Science Innovation Center, University of Fukui, Fukui, Japan

**Keywords:** early life stress, social isolation, social behavior, anxiety, prefrontal cortex, transcriptome

## Abstract

Early life stress (ELS), such as abuse, neglect, and maltreatment, exhibits a strong impact on the brain and mental development of children. However, it is not fully understood how ELS affects social behaviors and social-associated behaviors as well as developing prefrontal cortex (PFC). In this study, we performed social isolation on weaned pre-adolescent mice until adolescence and investigated these behaviors and PFC characteristics in adolescent mice. We found the ELS induced social impairments in social novelty, social interaction, and social preference in adolescent mice. We also observed increases of anxiety-like behaviors in ELS mice. In histological analysis, we found a reduced number of neurons and an increased number of microglia in the PFC of ELS mice. To identify the gene associated with behavioral and histological features, we analyzed transcriptome in the PFC of ELS mice and identified 15 differentially expressed genes involved in transcriptional regulation, stress, and synaptic signaling. Our study demonstrates that ELS influences social behaviors, anxiety-like behaviors through cytoarchitectural and transcriptomic alterations in the PFC of adolescent mice.

## Introduction

The environment is a crucial factor for providing optimal growth and health conditions for children, including social, cognitive, or immune system-related aspects (Ferguson et al., 2013; Sonuga-Barke et al., 2017; Consiglio and Brodin, 2020; Mackes et al., 2020). Particularly, childhood environments have great impacts on the brain architecture, synaptic plasticity, and mental development (Takesian and Hensch, 2013; Miguel et al., 2019).

For example, adverse childhood environments and experiences such as abuse, neglect, and maltreatment have profound effects on the brain and mental development, and constitutes major risk factors for brain structural changes and adult psychopathology (Teicher et al., 2016; Jaffee, 2017). Human brain imaging studies have reported that maltreatment reduces specific brain region volumes such as anterior cingulate cortex, ventromedial and dorsomedial PFC, hippocampus, and striatum, and these brain regions are highly involved in emotional control and the reward system (Chugani et al., 2001; Cohen et al., 2006; Edmiston et al., 2011; Dannlowski et al., 2012; Teicher et al., 2012; Kelly et al., 2013; Teicher et al., 2016). Importantly, specific brain regions are affected by specific types of abuse, neglect or maltreatment. Exposure to harsh corporal punishment caused a reduced gray matter volume in the prefrontal cortex of young adults (Tomoda et al., 2009). In addition, exposure to parental verbal aggression in childhood reduced fractional anisotropy in the arcuate fasciculus, and affected the development of the auditory association cortex involved in language processing (Tomoda et al., 2011). The child brain development has plasticity, and the effects of the childhood environments and experiences lead to structural and functional changes in the brain, as well as behavioral alterations.

Social isolation, social defeat stress, and electric shock are widely used as animal models to reproduce ELS and traumatic experiences in childhood (Schöner et al., 2017; Aspesi and Pinna, 2019; Pinna, 2019). A previous study of animal models reported that socially isolated mice during postnatal days (P) 21–35 displayed reduced sociality, working memory, and myelination in the PFC (Makinodan et al., 2012). In addition, reduced synapses and endogenous excitatory subtypes of the layer 5 pyramidal neurons were reported in the PFC of mice socially isolated for the same period (Yamamuro et al., 2018). We have also reported that social isolation during P21–53 induced the alteration of gut microbiota in mice (Usui et al., 2021b). These studies indicate that social isolation causes a lack of social experience and isolation stress, resulting in serious impacts on both PFC architecture and physical health. However, it is not fully understood how ELS affects various social behaviors and its-associated behaviors as well as transcriptome in the PFC of stressed individuals.

To address these questions, we performed social isolation on weaned pre-adolescent mice until adolescence for analyzing the impacts of ELS. In this study, we investigated various social behaviors (interaction, novelty, and preference), anxiety-like behaviors as well as cytoarchitecture and gene expressions by RNA sequencing (RNA-seq) of the PFC in adolescent ELS mice.

## Materials and Methods

### Mice

Wild-type C57BL/6N (Japan SLC Inc., Shizuoka, Japan) mouse was used. Mice were housed in the barrier facilities of University of Fukui under 50% humidity and a 12:12 h light:dark cycle (8:00 to 20:00) and given free access to water and food. Social isolation-induced ELS was performed as described previously (Usui et al., 2021b). Briefly, the used experimental and control mice came from the litters of the same mother. For ELS (*via* social isolation), male mice were individually housed using a filter cap (#CL-4150, CLEA Japan, Inc., Tokyo, Japan) immediately after the weaning at P21 until P50. For controls, 4 male mice were co-housed in the same cage without filter cap. Mice behaviors were assessed from P50 and kept in each housing condition until dissection at P67. A total of 39 mice were used in this study, using littermates that gave birth to 6 or more at a time. The number of mice used in each experiment is shown in corresponding figure legend. All procedures were performed according to the ARRIVE guidelines and relevant official guidelines under the approval (#27-010) of the Animal Research Committee of the University of Fukui. All behavioral tests were performed between 10am and 5pm. Experimenters blind to housing information performed all behavioral tests.

### Three-Chamber Social Interaction Test

Three-chamber social interaction test was performed as described previously (Usui et al., 2021b). Sociability and social novelty behaviors were assessed using a three-chamber box (W610 × D400 × H220 mm) with an infrared video camera system (O’Hara & Co., Ltd, Tokyo, Japan) at P53 after collecting fecal samples. For the first trial, empty wired cages were placed into both chambers for habituation. For the second trial, a stranger male mouse (mouse A) was placed into a wired cage of the right chamber to examine the sociability. For the third trial, mouse A was kept in the same wired cage as a familiar mouse, and a novel stranger male mouse (mouse B) was placed into a wired cage of the left chamber to examine the social novelty. Each trial was examined for 5 min after which the interactions with the targets were scored using an infrared video camera system (O’Hara & Co., Ltd, Tokyo, Japan).

### Social Interaction Test

Two mice were placed in the diagonal corners of a novel chamber (W480 × D480 × H330 mm) and allowed to examine the social interactions for 10 min. Time spent in social interaction was measured and tracked by digital counters with infrared sensors (SCANET MV-40, MELQUEST Ltd., Toyama, Japan).

### Social Preference Test

Mice were placed in one of the corners of a novel chamber (W480 × D480 × H330 mm) and allowed to examine the social preference for 10 min. A stranger male mouse was placed into a wired cage (80 × 80 mm) of the center of arena to examine the social preference. Time spent around the wired cage area (250 × 250 mm) and locomotor activity were measured and tracked by digital counters with infrared sensors (SCANET MV-40, MELQUEST Ltd., Toyama, Japan).

### Light-Dark Box Test

Mice were placed in the dark room of a light -dark box (W300 × D150 × H150 mm) and allowed to freely explore for 10 min. Time spent in each box (W150 × D150 × H150 mm), transition, and locomotor activity were measured and tracked by digital counters with infrared sensors (SCANET MV-40, MELQUEST Ltd., Toyama, Japan).

### Open Field Test

Mice were placed in one of the corners of a novel chamber (W480 × D480 × H330 mm) and allowed to freely explore for 120 min. Time spent in the center of arena (80 × 80 mm) and locomotor activity were measured and tracked by digital counters with infrared sensors (SCANET MV-40, MELQUEST Ltd., Toyama, Japan).

### Marble-Burying Test

Marble-burying test was performed as described previously (Usui et al., 2021a). Mice were placed in the corner of a novel home cage evenly placed eighteen marbles and allowed to freely explore for 20 min. After 20 min, the number of marbles buried was recorded. A marble was defined as buried when less than one-third of the marble was visible.

### Immunohistochemistry

Immunohistochemistry was performed as described previously (Usui et al., 2021a). The mouse brain was fixed with 4% PFA in PBS overnight at 4°C, cryoprotected in 30% sucrose in PBS overnight at 4°C, and then embedded in Tissue-Tek O.C.T. Compound (#4583; Sakura Finetek Japan Co., Ltd., Osaka, Japan) for cryosectioning. Cryosections (20 μm thick) were placed in PBS and then permeabilized in PBS-T for 30 min at room temperature. Blocking was performed using 10% goat serum and 1% BSA in PBS-T for 1 h at room temperature. Sections were stained with the following established primary antibodies overnight at 4°C: mouse monoclonal anti-NeuN (1:200, #MAB377, Millipore, Billerica, MA), rabbit polyclonal anti-Iba1 (1:1000, #019-19741, FUJIFILM Wako pure chemical corporation, Osaka, Japan). Sections were washed three times with PBS-T, incubated with species-specific antibodies conjugated to Alexa Fluor 488 (1:2,000; Invitrogen, Carlsbad, CA, United States) for 1 h at room temperature, and then washed 3 times with PBS-T. Cover glasses were mounted with Fluoromount/Plus (#K048; Diagnostic BioSystems, Pleasanton, CA, United States) or ProLong Diamond Antifade Mountant with DAPI (#P-36931 or #P36971, Thermo Fisher Scientific, Waltham, MA, United States) for nuclear staining. DAPI (#11034-56; Nacalai Tesque, Kyoto, Japan) was also used to stain nuclei. Images were collected using an all-in-one fluorescence microscope (BZ-X700, KEYENCE Corporation). Immunopositive cells in the PFC (cingulate and prelimbic cortical regions) were manually quantified within the area (4.8 × 10^5^ μm) at bregma 1.98 to 1.78 by an experimenter blind to housing information.

### RNA-Sequencing

RNA-seq was performed as a service by Bioengineering Lab. Co., Ltd. (Kanagawa, Japan). Briefly, total RNA was extracted with the AllPrep DNA/RNA Mini Kit (#80204, Qiagen, Hilden, Germany) according to the manufacturer’s instruction. RNA integrity number (RIN) of total RNA was quantified by Agilent 2100 Bioanalyzer using Agilent RNA 6000 Pico Kit (#5067-1513, Agilent, Santa Clara, CA). Total RNA with RIN values of ≥8.8 were used for RNA-seq library preparation. mRNA was purified from 500 ng total RNA by KAPA mRNA Capture Kit (#KK8440, Kapa Biosystems, Boston, MA), and subjected to cDNA library making (fragmentation, first and second strand syntheses, adenylation, ligation and amplification) using KAPA Stranded mRNA-Seq Kit (#KK8400, Kapa Biosystems, Boston, MA) according to the manufacturer’s instruction. cDNA library quality was quantified by 2100 Bioanalyzer using Agilent High Sensitivity DNA Kit (#5067-4626, Agilent, Santa Clara, CA). Library was sequenced as 151 bp paired-end on Illumina NextSeq 500.

### RNA-Seq Data Analysis

RNA-seq data analysis was performed as described previously (Usui et al., 2017a). Raw reads were first filtered for quality and trimmed for adapters using FASQC (http://www.bioinformatics.babraham.ac.uk/projects/fastqc) and Trimmomatic (Bolger et al., 2014). Filtered reads were then aligned to the mouse genome mm10 (https://genome.ucsc.edu) using STAR v2.5.2b (Dobin et al., 2013) allowing three mismatches. Uniquely mapped reads (bam flag NH:i:1) were used to obtain the gene counts using HTSeq package (Anders et al., 2015), and the read counts were normalized to the CPM (counts per million) implemented in the edgeR package (Robinson et al., 2010; McCarthy et al., 2012). For further analysis, we performed a sample-specific CPM filtering considering genes with CPM values of 1 in all replicates for treatments or controls. DESeq (Anders and Huber, 2010; Love et al., 2014) was then used to detect the differentially expressed genes (DEGs). We applied a filter of FDR (adjusted *p*-value) of <0.05 and absolute log fold change of >0.3 to identify significantly changed genes. Gene ontology (GO) analysis with the significant DEGs was carried out using ToppGene (https://toppgene.cchmc.org), and these GO terms were consolidated using REVIGO (Supek et al., 2011). The list of autism spectrum disorder (ASD) genes was derived from the SFARI database (1010 genes; https://gene.sfari.org/database/human-gene). The NCBI Gene Expression Omnibus (GEO) accession number for the RNA-seq data reported in this manuscript is GSE180055 (token: ivureikyjdwthuf).

### Quantitative Real-Time PCR

qPCR was performed as described previously (Usui et al., 2017b). Total RNA was extracted from the mouse PFC using the miRNeasy Mini Kit (#217004; Qiagen, Hilden, Germany) according to the manufacturer’s instructions. Single-stranded cDNA was prepared using DNaseI, Amplification grade (#18068015; Thermo Fisher Scientific) and SuperScript III First-Strand Synthesis SuperMix (#18080400; Thermo Fisher Scientific) and amplified by PCR according to the manufacturer’s instructions. qRT-PCR was performed using PowerUp SYBR Green Master Mix (#A25742; Thermo Fisher Scientific) and a QuantStudio 7 Flex Real-Time PCR System (Thermo Fisher Scientific). Each biological sample had four technical replicates for qPCR, and the number of biological replicates for each experiment is indicated in each figure legend. *18S* rRNA was used as a reference for normalization. Data were analyzed by the ΔΔCq method using QuantStudio 7 Flex Real-Time PCR System software (Thermo Fisher Scientific). The following primers were used: *18S rRNA*, F-5′-GAGGGAGCCTGAGAAACGG-3′, R-5′-GTCGGGAGTGGGTAATTTGC-3′; *mZbtb16*, F-5′-AGGGAGCTGTTCAGCAAGC-3′, R-5′-TCATCCCACTGTGCAGTTTC-3′; *mBanp*, F-5′-GTGGTTCAGATTGCAGTGGA-3′, R-5′-TCAAGGCAGGTTCATCATCA-3′; *mHif3a*, F-5′-GAGTGGAACCAGGTGGAAAA-3′, R-5′-TCTTGGTCACAGGGATGGAT-3′; *mNr4a1*, F-5′-CAATGCTTCGTGTCAGCACT-3′, R-5′-TGGCGCTTTTCTGTACTGTG-3′; *mPer2*, F-5′-GCGGTCACGTTTTCCACTAT-3′, R-5′-CTTGGGGAGAAGTCCACGTA-3′; *mFos*, F-5′-ATGGGCTCTCCTGTCAACAC-3′, R-5′-TGTCACCGTGGGGATAAAGT-3′; *mMcf2l*, F-5′-TGGCCCTAGAAGAAATGGTG-3′, R-5′-TTCTTCAGCTGCTCCTGGAT-3′; *mTsc22d3*, F-5′-GGTGGCCCTAGACAACAAGA-3′, R-5′-TCTTCTCAAGCAGCTCACGA-3′; *mLrfn2*, F-5′-GGTCAAGTGGTCTGTCAGCA-3′, R-5′-TAGCCAGTACGCACAAGTCG-3′; *mOsbpl3*, F-5′-GGAATACAGCGAGCTTCTGG-3′, R-5′-TGCATTCGTACGTTTCTCCA-3′; *mFkbp5*, F-5′-CCCCAATGCTGAGCTTATGT-3′, R-5′-TCCCTTCTCTTTCACGATGG-3′; *mSh2d3c*, F-5′-GGCGAAGCATTACCATGACT-3′, R-5′-TGGAGATCTGGGATCTGGTC-3′; *mArc*, F-5′-AGCCAGGAGAATGACACCAG-3′, R-5′-CCAGGCACCTCCTCTCTGTA-3′; *mNpas4*, F-5′-GGACTTGGCCACCTATGAAA-3′, R-5′-GCTCCACGTCTTGATGACAA-3′; *mCys1*, F-5′-CTACGCCTGCTGGATCAGTT-3′, R-5′-ACAGCTGTCTTCAGGGTTGC-3′. Cycling conditions were 50°C for 2 min and 95°C for 2 min, followed by 40 cycles at 95°C for 1 s and 60°C for 30 s according to the manufacturer’s instructions.

### Statistical Analysis

All behavioral data are represented as the means of biologically independent experiments with ± standard errors of the mean (SEM). The statistical analyses (independent samples *t*-test and Pearson’s r) were performed using the GraphPad Prism 9 software. Asterisks indicate *p*-values (****p < 0.0001, ***p < 0.001, **p < 0.01, *p < 0.05) with *p* < 0.05 being considered as the threshold of statistical significance.

## Results

### ELS Caused Adolescent Social Impairments

**FIGURE 1:**
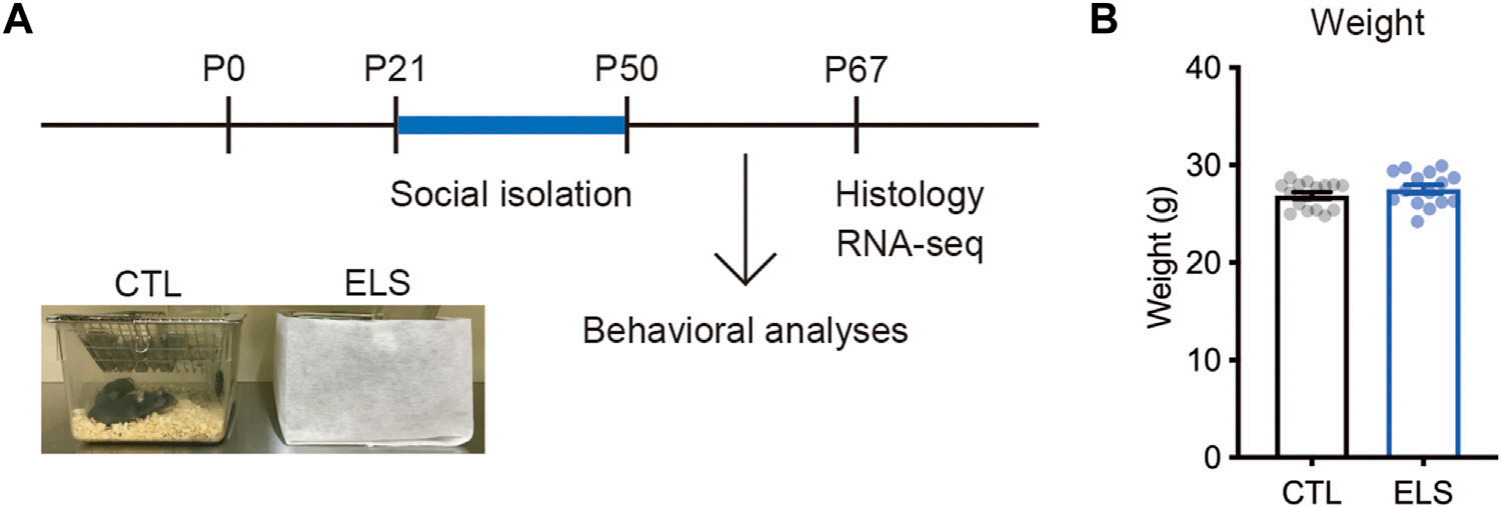
Experimental scheme. **(A)** Experimental design and time course of early life stress (ELS). In ELS mice, male mice were individually housed after the weaning at postnatal days 21 (P21) until P50. In control mice, 4 male mice were co-housed in the same cage after the weaning at P21. Mouse behaviors, brain, and gene expression were analyzed at P50 and P67, respectively. **(B)** No significant difference in the weight after social isolation. Data are presented as means (±SEM), unpaired *t*-test, *n* = 15–16/condition. CTL, control mice; ELS, early life stressed mice.

To investigate the influence of ELS on behaviors, cytoarchitecture, and transcriptome in the PFC of adolescent mice, we performed social isolation immediately after the weaning (Figure 1A). After the social isolation period (P21–P50), we found that there was no difference in the weight between controls and ELS mice (control = 26.89 ± 0.34, ELS = 27.55 ± 0.45, 95 %Cl = −0.4800 to 1.786, *p* = 0.25) (Figure 1B).

**FIGURE 2:**
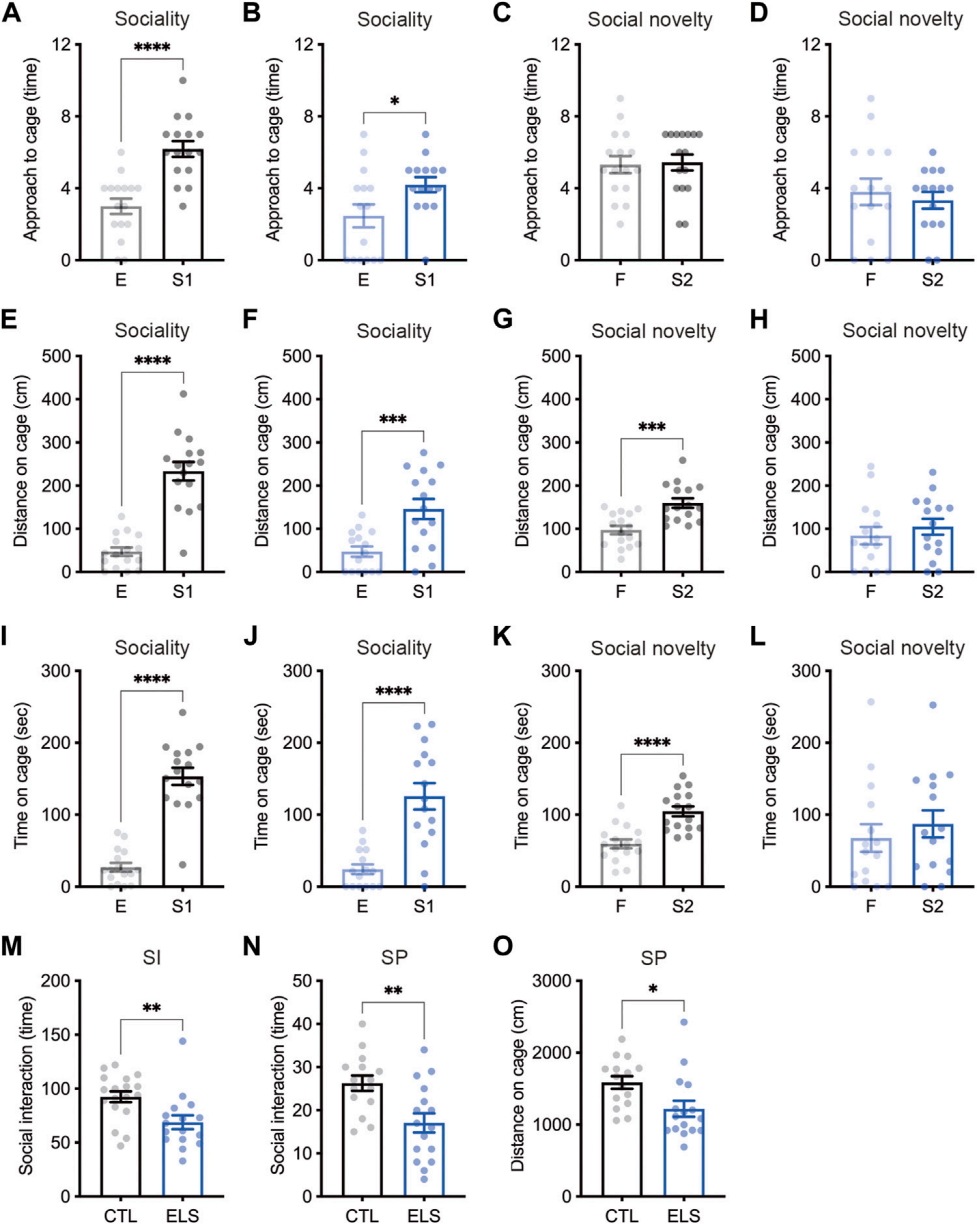
Social impairments induced by ELS. **(A–L)** Quantifications of three-chamber social interaction test results. The numbers of approaches **(A–D)**, distance **(E–H)**, and time **(I–L)** were shown during the 2nd trial (sociability behavior) **(A, B, E, F, I, J)** and the 3rd trial (social novelty behavior) **(C, D, G, H, K, L)** of three-chamber social interaction test (3CSI), respectively. Sociability and social novelty were impaired in adolescent ELS mice. CTL was shown in black, and the ELS was shown in blue. E, empty; S1, stranger mouse 1; S2, stranger mouse 2; SI, social interaction test. **(M)** Reduced number of social interactions in adolescent ELS mice. **(N, O)** Quantifications of social preference in ELS mice. The number of interaction **(N)** and distance **(O)** were reduced in ELS mice. SP, social preference test. Data are presented as means (±SEM). Asterisks indicate ****p < 0.0001, ***p < 0.001, **p < 0.01, *p < 0.05, unpaired *t*-test, *n* = 15–16/condition for 3CSI and SP, *n* = 16–18/condition for SI.

We examined social behaviors by multiple evaluation tests. In the sociability trial of three-chamber social interaction test, control and ELS mice interacted with a social target (stranger 1) in approach (controls: empty = 3.00 ± 0.43, stranger 1 = 6.19 ± 0.44, 95 %Cl = 1.934 to 4.441, *p* < 0.0001; ELS mice: empty = 2.47 ± 0.64, stranger 1 = 4.20 ± 0.42, 95 %Cl = 0.1712 to 3.295, *p* = 0.0309) (Figures 2A,B), distance (controls: empty = 47.08 ± 9.68, stranger 1 = 233.50 ± 21.56, 95 %Cl = 138.2 to 234.7, *p* < 0.0001; ELS mice: empty = 47.18 ± 11.98, stranger 1 = 146.10 ± 23.30, 95 %Cl = 45.22 to 152.6, *p* = 0.0008) (Figures 2E,F), and time (controls: empty = 27.09 ± 5.97, stranger 1 = 153.20 ± 11.92, 95 %Cl = 98.90 to 153.4, *p* < 0.0001; ELS mice: empty = 24.17 ± 6.79, stranger 1 = 125.60 ± 18.37, 95 %Cl = 61.27 to 141.5, *p* < 0.0001) (Figures 2I,J). However, ELS mice showed fewer interactions compared with control mice (approach: 95 %Cl = −3.230 to −0.7452, *p* = 0.0028; distance: 95 %Cl = −152.3 to −22.63, *p* = 0.0099; time: 95 %Cl = −71.89 to 16.58, *p* = 0.21) (Supplementary Figure S1), indicating the reduction of sociability in ELS mice. In the social novelty trial, control mice displayed more interactions with stranger mouse (stranger 2) than familiar mouse in approach (familiar = 5.31 ± 0.47, stranger 2 = 5.44 ± 0.45, 95 %Cl = −1.202 to 1.452, *p* = 0.85) (Figure 2C), distance (familiar = 97.04 ± 9.61, stranger 2 = 159.5 ± 10.98, 95 %Cl = 32.68 to 92.29, *p* = 0.0002) (Figure 2G), and time (familiar = 59.63 ± 6.08, stranger 2 = 104.70 ± 6.86, 95 %Cl = 26.31 to 63.75, *p* < 0.0001) (Figure 2K). In contrast, ELS mice showed no different interactions between a stranger mouse and a familiar mouse in approach (familiar = 3.80 ± 0.73, stranger 2 = 3.33 ± 0.47, 95 %Cl = −2.242 to 1.309, *p* = 0.59) (Figure 2D), distance (familiar = 84.09 ± 20.02, stranger 2 = 104.80 ± 18.65, 95 %Cl = 35.30 to 76.80, *p* = 0.46) (Figure 2H), and time (familiar = 67.63 ± 19.05, stranger 2 = 87.20 ± 18.78, 95 %Cl = −35.22 to 74.35, *p* = 0.47) (Figure 2L). These results indicate that sociability and social novelty are both impaired in adolescent ELS mice.

We next examined social interaction under the free moving condition in the chamber, and found the reduced social interaction events in ELS mice (control = 92.44 ± 5.05, ELS = 68.88 ± 6.39, 95 %Cl = −39.99 to −7.145, *p* = 0.0063) (Figure 2M). We also found that the reductions of social interaction events (control = 26.27 ± 1.78, ELS = 17.06 ± 2.23, 95 %Cl = −15.09 to −3.314, *p* = 0.0034) (Figure 2N) and distance around social target (control = 1586 ± 87.56, ELS = 1219 ± 111.1, 95 %Cl = −659.0 to −75.29, *p* = 0.0155) (Figure 2O) in ELS mice by social preference test. Together, these results demonstrate that various social behaviors are impaired in adolescent ELS mice.

### Anxiety-Like Behavior Induced by ELS

**FIGURE 3:**
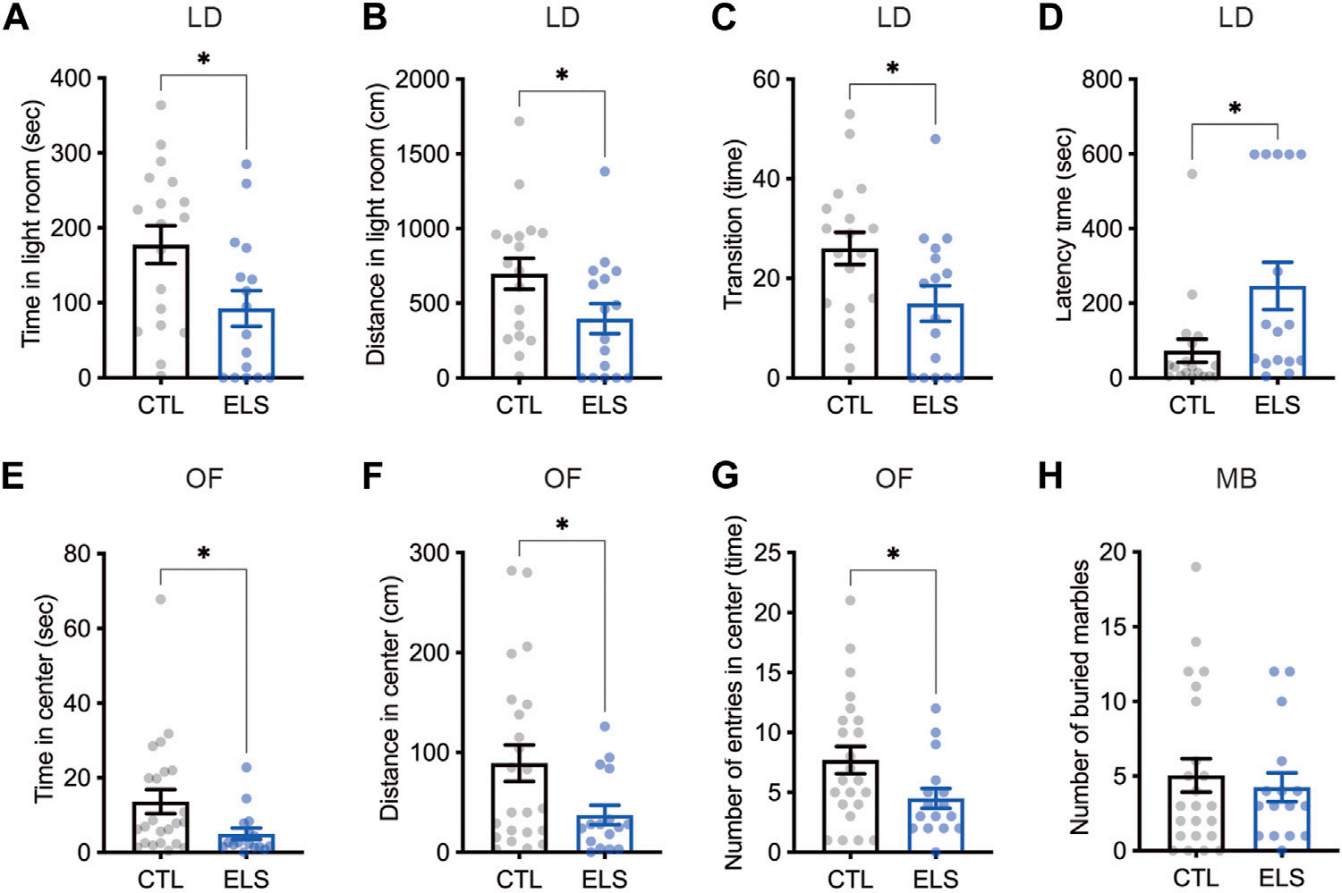
Increased anxiety-like behaviors in ELS mice. **(A–D)** Quantifications of light-dark box test result. Significant reductions of time **(A)** and distance **(B)** in light room, the transition number between the rooms **(C)**, and latency time to enter the light room **(D)** were observed in adolescent ELS mice. LD, light-dark box test. **(E–G)** Quantifications of open field test result. Significant reductions of time **(E)**, distance **(F)**, and the number of entries **(G)** in center were observed in adolescent ELS mice. **(H)** Quantification of the number of buried marbles. There was no difference in the repetitive behavior in adolescent ELS mice. MB, marble-burying test; OF, open field test. Data are presented as means (±SEM). Asterisk indicates *p < 0.05, unpaired *t*-test, *n* = 16–18/condition for LD, *n* = 16–23/condition for OF, *n* = 16–23/condition for MB.

Since the ELS mice showed impaired social behaviors, we investigated anxiety-like behaviors as social-associated behaviors. In light-dark box test, we found that decreased time (control = 177.50 ± 25.29, ELS = 92.46 ± 23.84, 95 %Cl = −156.4 to −13.75, *p* = 0.0209) (Figure 3A) and distance (control = 696.90 ± 104.00, ELS = 397.10 ± 100.10, 95 %Cl = −595.7 to −4.104, *p* = 0.0471) (Figure 3B) of the light room in ELS mice. We also found that decreased the number of transitions between the rooms (control = 26.00 ± 3.26, ELS = 14.94 ± 3.53, 95 %Cl = −20.84 to −1.285, *p* = 0.0278) (Figure 3C) and increased latency time (control = 73.24 ± 30.97, ELS = 246.2 ± 63.68, 95 %Cl = 33.67 to 312.2, *p* = 0.0166) (Figure 3D) in ELS mice. In addition, decreased time (control = 13.63 ± 3.22, ELS = 4.99 ± 1.50, 95 %Cl = −16.90 to −0.3771, *p* = 0.0409) (Figure 3E) and distance (control = 89.2 ± 18.19, ELS = 37.4 ± 9.68, 95 %Cl = −99.05 to −4.552, *p* = 0.0325) (Figure 3F) in the center area of open field test, and the number of entries to the center area (control = 7.70 ± 1.14, ELS = 4.50 ± 0.82, 95 %Cl = −6.291 to −0.1001, *p* = 0.0434) (Figure 3G) were observed in ELS mice. We also examined repetitive behavior of ELS mice by marble-burying test, however there was no difference (control = 5.04 ± 1.11, ELS = 4.25 ± 0.96, 95 %Cl = −3.951 to 2.364, *p* = 0.61) (Figure 3H). These results indicate that anxiety-like behavior is increased in ELS mice.

### Neuronal Loss and Microglia Increase by ELS

**FIGURE 4:**
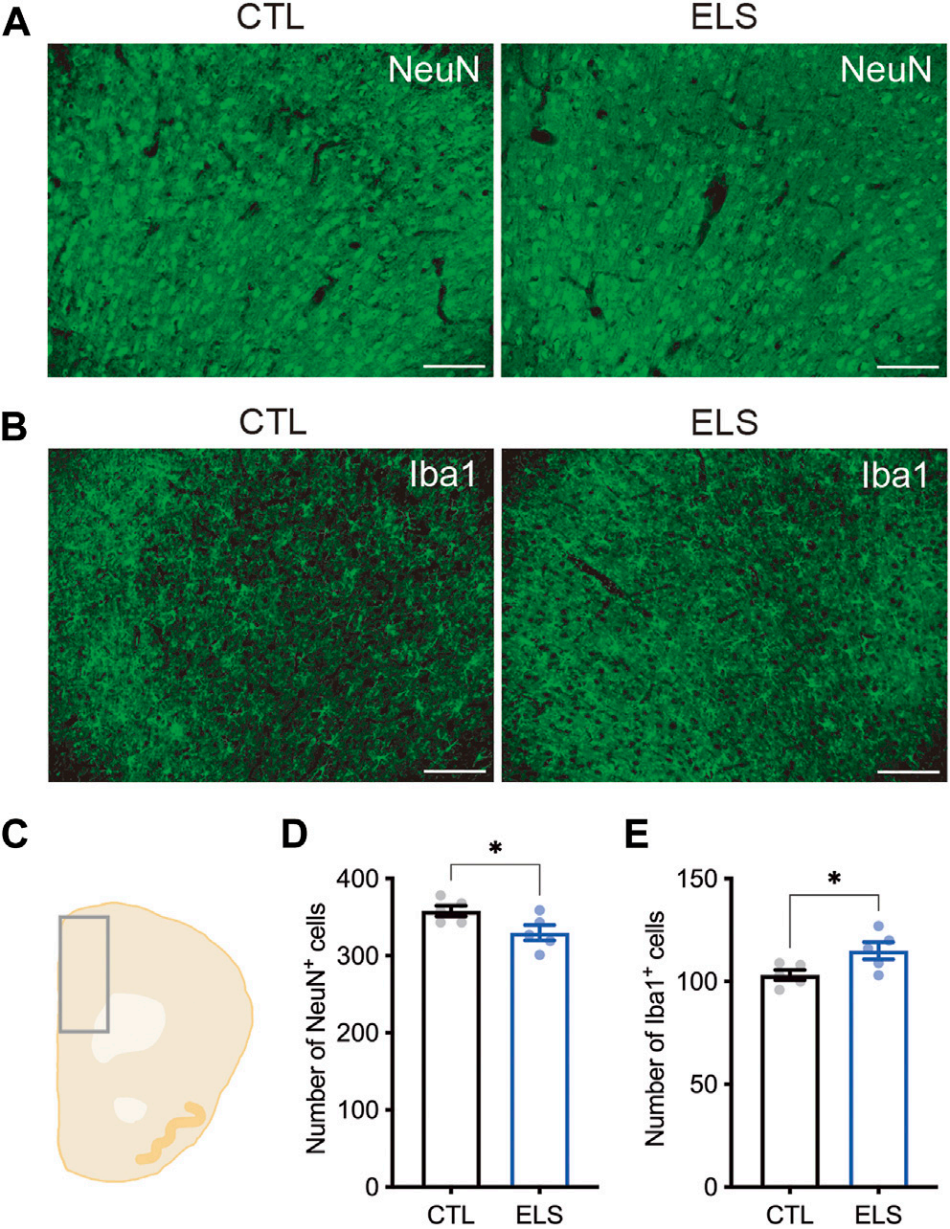
Neuronal loss and microglia increase in the PFC of ELS mice. **(A, B)** Representative fluorescent images of NeuN+ neuron **(A)** and Iba1+ microglia **(B)**. **(C)** Schematic diagram of mouse cortex analyzed at bregma 1.94. **(D, E)** Quantifications of NeuN+ neuron **(D)** and Iba1+ microglia **(E)** in the PFC. Decreased NeuN+ neurons and increased Iba1+ microglia were observed in the PFC of adolescent ELS mice. Data are presented as means (±SEM). Asterisk indicates *p < 0.05, unpaired *t*-test, *n* = 5/condition. Scale bars: 100 μm.

Next, we examined how ELS affects the PFC cytoarchitecture. We found a slight reduction of the number of NeuN+ mature neurons in the PFC of ELS mice (control = 358.00 ± 6.89, ELS = 329.80 ± 9.95, 95 %Cl = −56.12 to −0.2850, *p* = 0.0482) (Figures 4A,C,D). We found a slight increase of the number of Iba1+ microglia in the PFC of ELS mice (control = 103.20 ± 2.44, ELS = 115.00 ± 4.18, 95 %Cl = 0.6355 to 22.96, *p* = 0.0407) (Figures 4B,C,E). These results indicate that ELS caused a decrease in the number of neurons and an increase in the number of microglia in the PFC of adolescent ELS mice.

### ELS Altered PFC Transcriptome

**FIGURE 5:**
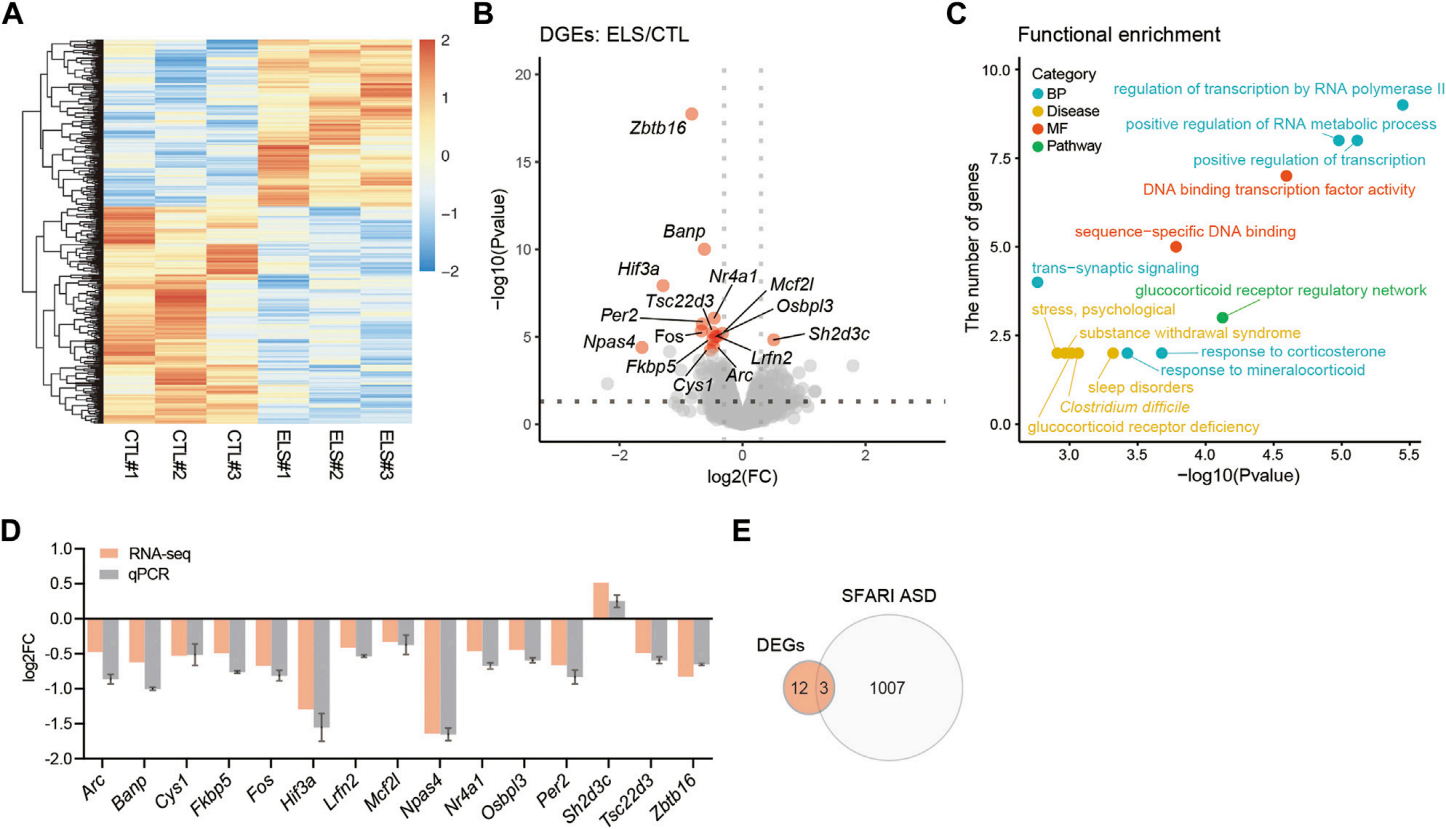
Transcriptomic alteration in the PFC of ELS mice. **(A)** Heatmap of gene expressions in the PFC. **(B)** Volcano plots showing ELS differentially expressed genes (DEGs). FDR < 0.05. X-axis = −log10(Pvalue), Y-axis = log2(FC). FC, fold change. **(C)** Scatter plots represent functional enrichment of ELS DEGs in biological process (BP), disease, molecular function (MF), and pathway. X-axis = −log10(Pvalue), Y-axis = the number of genes. *p*-Values reported by FDR < 0.05 threshold. **(D)** qPCR confirmation of ELS DEGs expressions in the PFC. **(E)** Venn diagram of ELS DEGs overlapping with SFARI autism spectrum disorder (ASD) genes. The overlapping 3 genes are *Zbtb16, Per2,* and *Lrfn2*. *n* = 3/condition for RNA-seq.

To understand the molecular mechanisms underlying behavioral and histological phenotypes, we characterized the transcriptome in the PFC of ELS mice by RNA-seq. Transcriptome profiles were clearly separated between control and ELS mice (Figure 5A). Differential expression analysis of the RNA-seq data uncovered 15 DEGs (FDR < 0.05) in PFC of ELS mice compared to control mice (Figure 5B, Supplementary Table S1). GO analysis for the DEGs highlighted functions involved in regulation of transcription by RNA polymerase II, positive regulation of transcription, positive regulation of RNA metabolic process, response to corticosterone, response to mineralocorticoid, and trans−synaptic signaling (Figure 5C, Supplementary Table S2). DEGs were also involved in the pathway in glucocorticoid receptor regulatory network, and diseases such as sleep disorders, *Clostridium difficile*, glucocorticoid receptor deficiency, substance withdrawal syndrome, and stress, psychological (Figure 5C, Supplementary Table S2). The expressions of DEGs were confirmed in the PFC of ELS mice by qPCR (Figure 5D). Since ELS mice exhibited social impairments, we overlapped the DEGs with the gene list of ASD, a developmental disorder that shows impairments in social communication. Then, we found that 3 DEGs (*Zbtb16*, *Per2*, *Lrfn2*) overlapped with SFARI ASD genes (Figure 5E). Together, these results indicate that ELS affected genes are involved in transcriptional regulation, stress signaling, and sleep disorders. Our results also suggest that 3 DEGs (*Zbtb16*, *Per2*, *Lrfn2*) are involved in sociality.

## Discussion

In this study, we demonstrate that ELS causes the impairments of social behaviors (sociality, social novelty, and social preference) and an increased anxiety-like behavior in adolescent mice. ELS induced neuronal loss and microglia increase in the PFC of adolescent mice. As the ELS-affected genes, we identified 15 DEGs involved in transcriptional regulation, stress signaling, and sleep disorders in the PFC of adolescent mice.

Previous studies have reported that social isolation at pre-adolescent period caused the reduction of sociality in mice (Makinodan et al., 2012; Usui et al., 2021b). Here we showed that various social impairments and increased anxiety-like behaviors in ELS mice (Figures 2, 3). Social isolation during adulthood has been used as a well-known method of generating depression models, and such mice show increased anxiety-like behaviors (Du Preez et al., 2020). In fact, increased anxiety-like behavior, repetitive behavior, memory impairment, and depression-like behavior have been reported in mice that were socially isolated earlier (P14–50) than our condition (Niwa et al., 2011). Sociality and anxiety are closely related and are controlled by neural circuits centered on PFC and amygdala (Stein and Stein, 2008; Penninx et al., 2021). These two brain regions have been reported as ones of corresponding regions involved in social behaviors (Amaral et al., 2008), and are also known as the brain regions impaired by neglect and maltreatment (Teicher et al., 2016). In the PFC of ELS mice, we observed slight changes in the numbers of NeuN+ mature neurons and Iba1+ microglia (Figure 4). This result suggests the possibility that ELS may cause inflammation and results neuronal cell death in the brain. To support this idea, previous studies have reported neuronal cell death due to chronic stress (Bachis et al., 2008; Natarajan et al., 2017). Moreover, chronic stress has been reported to increase blood corticosterone and induce an increase in microglia (Du Preez et al., 2020). It will be interesting to analyze the effects of ELS on the amygdala as a future study.

In terms of gene expression in the PFC of ELS mice, we identify total 15 DEGs involved in transcriptional regulation, stress signaling, and sleep disorders (Figures 5B,C). It was reasonable that there were changes in the genes related to such GOs due to ELS. Among those DEGs, there were many transcription factors, suggesting ELS causes functional changes in PFC. The alterations of genes involved in stress signals were presumed to be the effects of chronic stress due to social isolation. It is known that sleep disorders coexist in social withdrawal (Rubin et al., 2009) and psychiatric disorders such as major depressive disorder (Goldstein and Walker, 2014; Otte et al., 2016). Our data suggests that the alterations of genes associated with circadian rhythm such as *Per2* and *Npas4* due to the influences of ELS may trigger sleep disorders.

In order to explore the gene involved in sociality, we overwrapped the DEGs with SFARI ASD gene list, and identified *Zbtb16*, *Per2*, and *Lrfn2* (Figure 5E). Zbtb16 (Plzf) is known as a transcription factor playing key roles in many biological processes such as stem cell proliferation, differentiation, apoptosis, chromatin remodeling, metabolism and immunity (Suliman et al., 2012; Šeda et al., 2017). *ZBTB16* is associated with ASD (Bacchelli et al., 2019) and skeletal defects, genital hypoplasia, and mental retardation (SGYMR) (Wieczorek et al., 2002; Fischer et al., 2008). *Zbtb16* KO mice displayed ASD-like behaviors such as social impairment and increased repetitive behaviors as well as cognitive impairment (Usui et al., 2021a). However, *Zbtb16* KO mice showed antianxiety-like (risk-taking) behaviors (Usui et al., 2021a). Per2 is known as a clock gene of Period family and controls circadian rhythm under the CLOCK/ARNTL regulation (Takahashi, 2017; Rijo-Ferreira and Takahashi, 2019). *PER2* is also associated with ASD (Iossifov et al., 2014; RK et al., 2017), and sleep problem has been reported to coexist in developmental disorders such as ASD (Couturier et al., 2005; Krakowiak et al., 2008; Souders et al., 2009; Mazurek and Sohl, 2016). Moreover, it has been reported that *Per2* expression was altered by social defeat stress (Moravcová et al., 2021). Lrfn2 (SALM1) is known as a synapse adhesion molecule interacting with PSD-95 (Ko et al., 2006). *LRFN2* is associated with ASD (Voineagu et al., 2011), learning disabilities (Thevenon et al., 2016), and antisocial personality disorder (Rautiainen et al., 2016). *Lrfn2* KO mice also displayed ASD-like behaviors such as social withdrawal, decreased vocal communications, increased repetitive behaviors and prepulse inhibition deficits, but not anxiety-like behaviors (Morimura et al., 2017). *Lrfn2* KO mice also displayed synaptic morphological abnormalities in the CA1 hippocampal neurons, resulting LTP enhancement (Morimura et al., 2017). Together, these findings suggest that *Zbtb16*, *Per2*, and *Lrfn2* are the genes involved in sociality.

We acknowledge that our study has several limitations. Since only male mice are used in this study, caution must be taken in interpreting the results of behavioral analysis. In addition, despite the analysis of the effects of environmental factors, the sample size in transcriptome analysis was small and the *p* value in gene expressions was not significant, thus only few genes were identified as DEGs. It is expected that increasing the sample size will lead to the identification of more genes affected by ELS. Furthermore, it is also important to understand the maternal gestational stress as a one of ELS, which is known to affect the brain development and behavior of the offspring. Previous studies have reported that maternal gestational stress has an epigenetic alteration on offspring gene expression as well as impairments of motor and cognitive development, immune system, and adaptation to stress (Wadhwa et al., 2001; Kingston et al., 2012; Argyraki et al., 2019).

In conclusion, our results demonstrate that ELS impacts adolescent social behaviors, anxiety-like behaviors, and PFC characteristics in mice. Our study suggests that ELS causes the alterations in the cytoarchitecture and transcriptome of PFC, leading to social impairments and increased anxiety-like behaviors in adolescents.

## Data Availability

The datasets presented in this study can be found in online repositories. The names of the repository/repositories and accession number(s) can be found in the article/Supplementary Material.

## Supplementary Material

Supplementary Figure S1 An impairment of sociality in ELS mice.

Supplementary Table S1 The list of DEGs in ELS mice.

Supplementary Table S2 The list of ToppGene information of DEGs.
