# Supplementary material for: Early Life Stress Alters Gene Expression and Cytoarchitecture in the Prefrontal Cortex Leading to Social Impairment and Increased Anxiety
Source: Front Genet. 2021 Nov 2;12:754198. doi: 10.3389/fgene.2021.754198 (PMC8593203; doi:10.3389/fgene.2021.754198)
Supplement: Supplementary file 3 [file Image1.pdf]

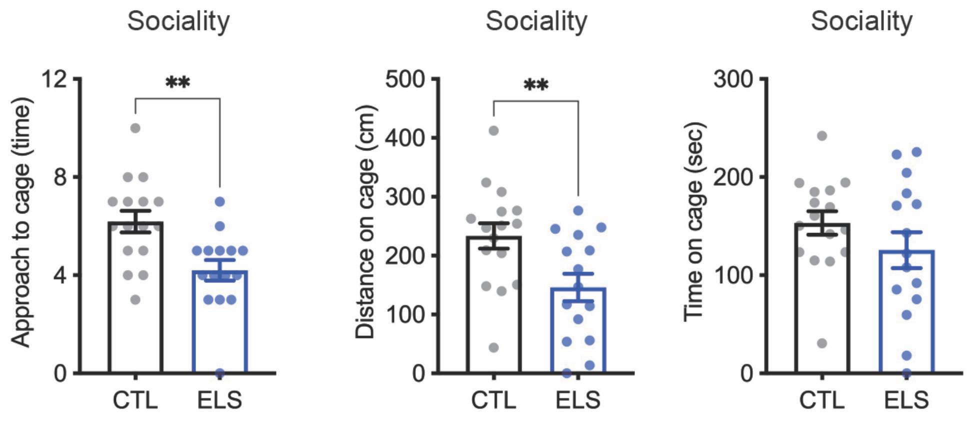

Supplementary Figure S1. An impairment of sociality in ELS mice.

Quantifications of three-chamber social interaction test results. The numbers of approaches, distance, and time were shown during the 2nd trial (sociability behavior) of three-chamber social interaction test (3CSI). Sociability was impaired in adolescent ELS mice. CTL was shown in black, and the ELS was shown in blue. Data are presented as means (±SEM). Asterisk indicates $^{**}P < 0.01$, unpaired *t*-test, n = 15-16/condition for 3CSI.
